# Caregiver dissatisfaction with their child’s participation in home activities after pediatric critical illness

**DOI:** 10.1186/s12887-020-02306-3

**Published:** 2020-09-02

**Authors:** Jessica M. Jarvis, Nora Fayed, Ericka L. Fink, Karen Choong, Mary A. Khetani

**Affiliations:** 1 Department of Occupational Therapy, College of Applied Health Sciences, University of Illinois at Chicago, 1919 W. Taylor Street, AHSB 316A, Chicago, IL 60612 USA; 2 Department of Physical Medicine and Rehabilitation, University of Pittsburgh, Pittsburgh, PA USA; 3 School of Rehabilitation Therapy, Queen’s University, Kingston, Ontario Canada; 4 Department of Critical Care Medicine, UPMC Children’s Hospital of Pittsburgh, Pittsburgh, PA USA; 5 Department of Pediatrics, McMaster University, Hamilton, Ontario Canada

**Keywords:** Pediatrics, Rehabilitation, Critical care, Participation, Caregivers, Outcomes

## Abstract

**Background:**

Pediatric critical care is often accompanied by a variety of functional impairments. Preliminary evidence suggests children’s participation in home activities has a slow trajectory post-pediatric intensive care unit (PICU) discharge, however, additional and more granular knowledge on specific problematic activities is needed to inform patient-centric rehabilitative care. The objectives of this study are to identify common home activities in which caregivers’ report dissatisfaction and to determine predictors of caregivers’ dissatisfaction with their child’s participation in home activities post-PICU discharge.

**Methods:**

Secondary analyses of data from a prospective cohort study, the Wee-Cover study, using a subsample of caregivers (*N* = 170) of children 1–17 years, admitted to a PICU ≥48 h with data on our primary outcome measure from at least one time point. Data were gathered at enrollment and at 3 and 6 months post-PICU discharge. Caregivers reported on their dissatisfaction with their child’s participation in home activities via the Participation and Environment Measure. Common activities were identified by plotting caregiver dissatisfaction for each activity pre-and post-PICU, reporting activities in which ≥50% of caregivers reported dissatisfaction with post-PICU, and assessing for significantly different dissatisfaction levels between time-points for each activity. Predictors of caregiver dissatisfaction were assessed using Poisson generalized estimated equation models.

**Results:**

There was variability in reported dissatisfaction across all activities; ≥50% of caregivers reported dissatisfaction with five activities, including getting clean, personal care management, and mealtime for younger children and household chores and homework for school-aged children and youth. Four activities had significantly higher caregiver dissatisfaction post-PICU: sleep (children < 5 years), homework, indoor play and games, and computer/video games (children ≥5 years). Home environmental support and the interaction of having participation-focused strategies with receiving PICU-based rehabilitation services were negatively associated with caregiver dissatisfaction. Increased caregiver stress and functional performance were associated with increased dissatisfaction.

**Conclusions:**

Individualized PICU-based rehabilitation services to determine family priorities and develop participation-focused strategies, specifically those increasing environmental supports within the home, may ease the family’s transition home post-PICU.

**Trial registration:**

ClinicalTrials.gov Identifier NCT02148081 05/28/2014.

## Highlights

- Caregivers report a variety of their child’s daily life activities in the home with which they are dissatisfied with post-PICU, highlighting the need for individualized rehabilitation services.
- Caregivers whose child received PICU-based rehabilitation services and were equipped with participation focused strategies were more satisfied with their child’s participation in daily life activities in the home.
- Specific types of home environmental factors are associated with increased caregiver satisfaction with their child’s participation in daily life activities in the home.

## Background

There is emerging evidence that pediatric critical care survivorship is frequently accompanied by long-lasting impairments in various domains of functioning, for both children and their families [1]. These acquired impairments in physical, cognitive, social, and emotional function following critical illness are collectively referred to as post-intensive care syndrome [2]. Recovery from post-intensive care syndrome is a complex, multi-faceted process, requiring personalized support by rehabilitation services [3, 4]. If not addressed, these impairments may have persistent, long-term consequences on children’s performance of daily tasks and their ability to participate in valued activities that comprise functioning and quality-of-life. For example, only 67% of children experience any recovery in participation of activities of daily living following critical illness [4], and children’s home participation does not change significantly for the first 6 months post-PICU discharge [5].

As pediatric critical care shifts its focus from mortality to survivorship, there is need to understand the outcomes that patients and caregivers deem important during recovery [6, 7]*.* Children’s participation in valued activities is a key patient-centric outcome of rehabilitation and is associated with increased quality of life [8–11]. While children who survive a critical illness experience some recovery in mobility and cognitive function in the 6-months post-PICU discharge, preliminary evidence suggests that they continue to experience difficulty in their participation in home activities. This, in turn, is a source of persistent caregiver burden and poor quality-of life [4, 5]. The specific areas of participation that are of greatest concern to these families, and salient factors associated with caregiver concern, remain unknown.

New patient-reported outcome measures of children’s participation permit valid and reliable characterization of caregiver dissatisfaction with their child’s participation, by indicating if they desire to see their child’s participation in an activity change, and information on environmental factors that may influence their child’s participation [12–14].

This information is crucial for tailoring appropriate family-centric rehabilitation interventions with this population. Therefore, the first objective of this study was to determine common caregiver priorities for their child’s participation in two ways: 1) reporting on home activities in which ≥50% of caregivers’ report dissatisfaction 3 months post-PICU discharge, and 2) identifying activities in which caregivers report significantly different dissatisfaction prior to PICU admission and 3 months post-PICU discharge. The second objective was to determine predictors of caregivers’ dissatisfaction with their child’s participation in home activities 6-months post-PICU discharge.

## Methods

### Population

This was a secondary analysis of data from Wee-Cover study, a bi-center, Canadian prospective cohort study of PICU survivors [4]. Children aged 1–17 years, admitted for at least 48 h to the PICU with organ dysfunction were enrolled. Additional details on the Wee-Cover cohort study and sample description are published elsewhere [4, 5]. All data in this study were collected as a part of the original study. For the purpose of this study, only caregivers with data on our primary outcome measure from at least one time point were included (*N* = 170 caregivers). Institutional research ethics approval was obtained for both study sites, and an ethics waiver was obtained prior to analyzing the de-identified data for this study.

### Measures

Measure selection for this study was based on the Family of Participation-Related Constructs, a contemporary participation-focused rehabilitation research framework [14, 15]. The decision to include time-varying variables (i.e., measures collected at enrollment and 3 and 6 months post-PICU discharge) was based on prior work identifying the need for longitudinal research in pediatric rehabilitation [16].

#### Caregiver dissatisfaction with participation

Caregiver dissatisfaction with their child’s participation in the home was assessed using the home section of the Participation and Environment Measures (PEM), a patient reported outcome measure for parents of children with and without disabilities. In the PEM, caregivers assess their child’s participation in activities across home, school, and community settings (e.g., personal care management, household chores). For each activity, caregivers denoted whether they desire to see a change in their child’s participation (yes/no), which was included in the measure design to capture caregiver satisfaction with their child’s current participation, with ‘yes, desire change’ responses indicating dissatisfaction [17].

The Participation and Environment Measure for Children and Youth (PEM-CY) [13] was administered to caregivers of children aged 5–17 years and the Young Children’s Participation and Environment Measure (YC-PEM) [12] was administered to caregivers of children between 1 and 4 years old. Data on participation were collected at three time points: study enrollment (reported on pre-PICU participation), and 3 and 6 months post-PICU discharge. Both versions of the PEM have acceptable internal consistency reliability and test-retest reliability [5]; internal consistency within our sample was also established for the desire for change scale at all time points for the PEM-CY (α = 0.80–0.87) and YC-PEM (α = 0.91–0.95).

For the first objective, item responses on pre-PICU and 3 months post-PICU were reported to ascertain significant changes in dissatisfaction in the first few months post-PICU and to identify common areas where caregivers report dissatisfaction with their child’s participation in the first few months post-PICU discharge. For the second objective, a composite score of caregiver dissatisfaction with their child’s participation at home 6 months post-PICU was calculated by summing the number of times caregivers reported ‘yes’ to ‘would you like your child’s participation to change in this activity’, then dividing by the total number of home-based activities and multiplying by 100 (range 0–100). Higher percentages indicate more caregiver dissatisfaction with their child’s home participation.

Data from the measure of caregiver dissatisfaction with participation were used for both study objectives.

#### Child, caregiver, and environmental factors related to caregiver dissatisfaction with participation

When characterizing caregivers’ dissatisfaction with their child’s participation, it is crucial to delineate specific child factors (e.g., a child’s functional task performance), caregiver factors (e.g., caregiver stress), and environmental factors (e.g., physical and sensory layout of the home) that influence caregiver dissatisfaction with their child’s participation in valued activities within the real-life context of the home and family [5, 14, 18, 19]. Data from measures of child, caregiver, and environmental factors were included as predictors for the second study objective.

### Child factors

#### Functional performance

Children’s performance of functional tasks was assessed using the daily activities domain of the Pediatric Evaluation of Disability Inventory - Computer Adaptive Test (PEDI-CAT), a patient reported outcome measure [20]. The domain of daily activities was used for this study, as prior research has established associations between the PEM home involvement scores and children’s PEDI-CAT normative scores for daily activities [5, 21]. PEDI-CAT daily activities normative scores from observations at 3 months and 6 months post-PICU discharge were entered into the model as a continuous and time-varying variable.

#### PICU rehabilitation services

Data on receipt of rehabilitation services during the child’s PICU admission were gathered prospectively. Data were dichotomized as ‘1’, receipt of rehabilitation if they received occupational therapy, physical therapy, and/or speech therapy during PICU admission, or “0”, received no rehabilitation services during PICU admission.

### Caregiver factors

#### Participation-focused strategy use

When completing the PEM assessment, caregivers reported on their current strategies to facilitate their child’s participation in activities. Data on strategy use were first independently screened by two study staff, to exclude cases that did not qualify as a strategy, i.e., was not a complete phrase or did not indicate a change made to support their child’s participation [18]. Discrepancies were resolved via discussion with a third research staff and the use of majority rule. Then, data on strategy use at 3 and 6 months post-PICU discharge were dichotomized as ‘1’, having one or more strategies, or ‘0’, having no strategies and included in the model as a time-varying variable.

#### Caregiver stress

The Pediatric Inventory for Parents was used to assess the stress that caregivers experienced related to caring for their child with an illness. The Pediatric Inventory for Parents is a patient reported outcome measure containing 42 items over four domains: 1) child medical care, 2) communication related to and with their child, 3) role functioning, and 4) emotional functioning [22]. For each item, caregivers reported the frequnecy of the stressful event and the degree of difficulty associated with the stressful event, on a 5-point scale, from 1 [not at all] to 5 [extremely] [22]. Stress difficulty scores at 3 months and 6 months post-PICU were entered into the model as a continuous and time-varying variable.

### Environmental factors

#### Home environmental support

When completing the PEM, caregivers evaluated the impact of home environmental features and resources on their child’s participation in activities, on a 3-point scale, from 1 [usually makes harder/usually no] to 3 [usually helps/usually yes/no impact].

For this study, composite scores were calculated to capture home environmental support with greater specificity across the following four sub-domains: 1) physical layout and sensory qualities of the home; 2) activity demands (i.e., physical, cognitive, and social demands of home activities); 3) attitudes and relationships (i.e., relationships with and attitudes of family members and professionals who care for the child at home); and 4) resources within the home (i.e., supplies, information, time, and money). Composite scores for each sub-domain were calculated by summing responses across the relevant items, dividing the sum by the maximum possible environmental score for that sub-domain, and then multiplying by 100 (range 0–100).

### Data analysis

SPSS 24.0 was used for analyses. Descriptive statistics were used to describe sample characteristics.

For the first objective, caregiver dissatisfaction with their child’s home participation across all home activities was graphed using spider plots by age group (1–4 years and 5–17 years) and by time (pre-PICU admission and 3 months post-PICU discharge). The four activities with the highest percentages of caregivers reporting dissatisfaction at 3 months post-PICU were identified. The time point of 3 months post-PICU was used to identify need within the first few months following PICU discharge. Next, caregiver dissatisfaction for each home activity area was compared between baseline (pre-PICU) and 3 months post-PICU discharge using Wilcoxon signed rank tests to assess for significant differences in dissatisfaction following critical illness. Wilcoxon signed rank tests were used as data was not normally distributed and scores were from the same sample.

For the second objective, our dependent variable was caregiver dissatisfaction with home participation at 3 and 6 months. Caregiver dissatisfaction followed a Poisson distribution, thereby supporting the use of a generalized estimated equation model for the Poisson regression. This model was chosen given its ability to handle missing data in longitudinal studies, thus affording maximum use of our dataset. A backwards stepwise approach was then used to build the model, with a 0.10 exit to delineate predictors of caregiver dissatisfaction. The following predictors were initially selected for model inclusion: child factors (age, receipt of PICU rehabilitation, functional performance), caregiver factors (level of stress difficulty and having participation-focused strategies), and home environmental supports (physical layout and sensory qualities, activity demands, attitudes and relationships, and resources). We controlled for caregiver dissatisfaction with their child’s home participation prior to their critical illness. We also included an interaction between caregivers having participation-focused strategies and their child receiving PICU rehabilitation services [18].

## Results

### Sample characteristics

Of this substudy sample, there were 154 participants with data on the primary outcome measure at baseline, 116 at 3 months post-PICU, and 109 at 6 months post-PICU. Our sample were caregivers of children who were primarily male (52%) and had a prior diagnosed chronic condition (71%), with median age of 7.2 years (IQR: 3.0–13.5), that experienced a median PICU length of stay of 7 days (IQR: 4–12), and of which 54% received rehabilitation services in the PICU (see Table 1). All child, caregiver, and environmental factors were compared between those with and without data 6 months post-PICU among those with baseline data (*N* = 59); caregiver dissatisfaction with their child’s participation was higher pre-PICU in those lost to follow-up than those with data at 6 months (35% versus 24%, *p* ≤ 0.05).

**Table 1:** Baseline participant characteristics

Characteristics, median (IQR)	*N* = 170
Age, years	7.16 (2.96–13.46)
Male	52%
PICU length of stay, days	7 (4–12)
Received rehabilitation in PICU	54%
Pre-existing chronic condition^a^	71%
Caregiver dissatisfaction pre-PICU	20% (0–50%)

*IQR* Interquartile range presented as Q1-Q3, *PICU* Pediatric intensive care unit; ^a^Pre-existing chronic condition refers to an underlying medical condition diagnosed prior to PICU admission

### Common activities in which caregivers report dissatisfaction

Prior to their child’s critical illness, caregivers reported desiring their child’s participation to change in 20% (IQR: 0–50%) of home-based activities. At 3 months and 6 months post-PICU, caregivers reported desiring their child’s participation to change in 31% (IQR: 10–60%) and 23% (IQR: 0–62%) of home-based activities, respectively.

**Fig. 1:**
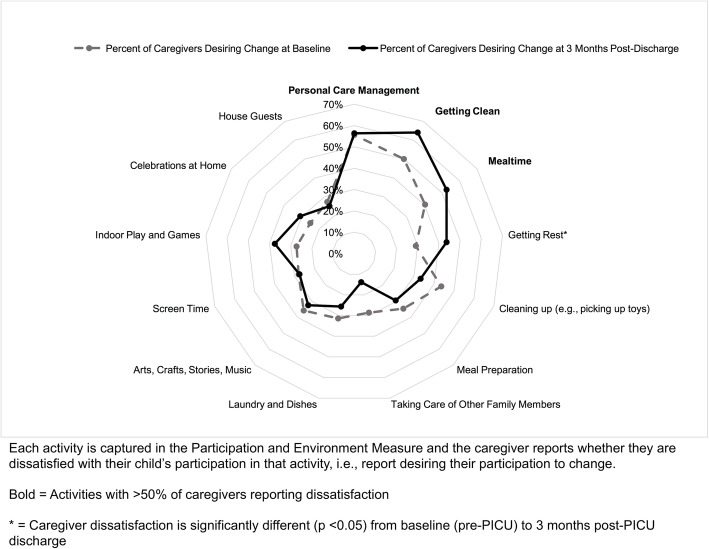
Percentage of caregivers dissatisfaction with their young child’s participation by home activity pre and post-PICU

As shown in Fig. 1, at 3 months post-PICU, the activities with ≥50% of caregivers reported dissatisfaction with for younger children ages 1–4 years old were getting clean (e.g., taking bath), personal care management (e.g., getting dressed), and mealtime (e.g., breakfast, snacks). Getting rest was the only activity within this age group with significantly different dissatisfaction from pre-PICU admission to 3 months post-PICU discharge, 29% vs. 44%, respectively (*p* ≤ 0.05).

**Fig. 2:**
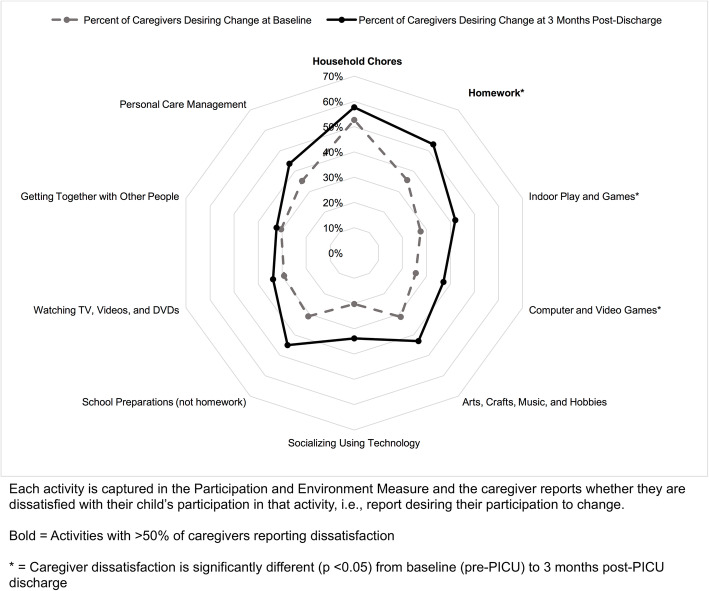
Percentage of caregivers reporting dissatisfaction with their school-aged child’s participation in home activities pre and post-PICU

As shown in Fig. 2, at 3 months post-PICU the activities with ≥50% of caregivers reported dissatisfaction with for school-aged children ages 5–17 years old were household chores (e.g., cleaning room, taking care of pet) and homework (e.g., homework assignments, school projects). Activities with significantly different caregiver dissatisfaction from pre-PICU to post-PICU were homework (36% vs 53%), indoor play and games (28% vs 42%), and computer and video games (26% vs 37%) (*p* ≤ 0.05).

### Child and caregiver factors predicting caregiver dissatisfaction with home participation

Data on the measurements of predictors of caregiver dissatisfaction with participation at 3 and 6 months post-PICU discharge are displayed in the Additional file 1.

#### Child and caregiver factors

The child’s functional performance of daily activities (*B* = 0.012, 95% CI = 0.009, 0.016) was significantly positively associated with caregiver dissatisfaction with their child’s participation in home-based activities (e.g., as a child’s functional performance increased, caregiver dissatisfaction with participation increased). Time since PICU discharge (*B* = − 0.095, 95% CI = − 0.145, − 0.044) and the interaction of caregivers’ having participation-focused strategies and the child receiving PICU rehabilitation services (*B* = − 0.548, 95% CI = − 0.676, − 0.421) were significantly negatively associated with caregiver dissatisfaction with their child’s participation in home-based activities. Caregiver stress difficulty (*B* = 0.003, 95% CI = 0.002, 0.004) and their dissatisfaction with their child’s participation pre-PICU admission (*B* = 0.016, 95% CI = 0.016, 0.017) were significantly positively associated with their dissatisfaction with their child’s participation in home-based activities.

#### Environmental factors

Home environmental supports with respect to attitudes and relationships (*B* = − 0.004, 95% CI = − 0.005, − 0.003), activity demands (*B* = − 0.001, 95% CI = − 0.002, − 0.001), and physical layout and sensory qualities (*B* = − 0.001, 95% CI = − 0.002, − 0.001) were significantly negatively associated with caregiver dissatisfaction with their child’s participation in home-based activities (e.g., when caregivers reported more environmental support, they also reported less dissatisfaction with their child’s participation) (see Table 2).

**Table 2:** Poisson model predicting caregiver dissatisfaction with home participation post-PICU

Variables,	*B* (95% CI)	*p*-value
Intercept	2.305 (2.024, 2.586)	< 0.001
Child Factors		
Time since PICU discharge	− 0.095 (− 0.145, − 0.044)	< 0.001
Functional performance	0.012 (0.009, 0.016)	< 0.001
Received PICU rehabilitation	0.714 (0.604, 0.824)	< 0.001
Caregiver Factors		
Pre-PICU dissatisfaction	0.016 (0.016, 0.017)	< 0.001
Caregiver has participation-focused strategy	0.269 (0.17, 0.368)	< 0.001
PICU rehabilitation* participation-focused strategy	−0.548 (−0.676, − 0.421)	< 0.001
Caregiver stress difficulty	0.003 (0.002, 0.004)	< 0.001
Home Environmental Factors		
Attitudes and relationships	−0.004 (− 0.005, − 0.003)	< 0.001
Activity demands	− 0.001 (− 0.002, − 0.001)	< 0.01
Physical layout and sensory qualities	−0.001 (− 0.002, − 0.001)	< 0.001

*PICU* Pediatric intensive care unit; Functional performance = Daily Activities domain of the Pediatric Evaluation of Disability Inventory – Computerized Adaptive Test; Caregiver stress difficulty = Pediatric Inventory for Parents; Caregiver dissatisfaction, participation-focused strategy, and home environmental factors (supports) = Participation and Environment Measure

## Discussion

Participation is as a key indicator of human health and well-being and is the ultimate objective of rehabilitation services [12]. Thus, as we work towards improving the long-term functional recovery of PICU survivors, it is crucial to build rehabilitation relevant knowledge regarding participation as a key patient-centered outcome [23]. Specifically, there is need for granular knowledge on areas of caregiver concern with their child’s participation and predictors of caregiver concern post-PICU discharge. Together, this foundational knowledge is required to design and test family-centric rehabilitative care for pediatric critical care survivors. This study found that 6 months post-PICU discharge, common areas of dissatisfaction related to personal care management, getting clean, mealtime, household chores and homework. Factors associated with caregiver dissatisfaction included home environmental supports, receipt of PICU-based rehabilitation services, and caregivers having participation-focused strategies among those whose child received PICU-based rehabilitation services.

The most common caregiver priorities were activities that children are required to do (i.e., non-discretionary). This finding is in contrast to prior work by Di Marino and colleagues, which found the most common activities caregivers of children with disabilities identified being dissatisfied with were activities such as screen time and socializing with friends [24]. When looking at activities with increased dissatisfaction following critical illness, there was a mix of non-discretionary activities (getting rest and homework) and discretionary, or leisure, activities (indoor play and games, computer and video games). Similarly, in a study comparing desire for participation change between caregivers of children with and without autism, the most commonly identified activity was non-discretionary (i.e., household chores) but at a much higher rate (i.e., at 91% and 80%, respectively) [25]. These differences in caregiver priorities for their children following critical illness is consistent with newly emerging research [3]. These findings highlight the importance of engaging with families during their PICU stay to ascertain their individualized priorities regarding recovery of their child and family life. Electronic-health tools have shown promise in reinforcing family-centered care in pediatric rehabilitation [26–28] and warrant testing within this population.

Caregivers reporting less dissatisfaction with their child’s participation from 3 to 6 months, as evident by the negative relationship between time since PICU discharge and caregiver dissatisfaction, was unanticipated as prior work with these data demonstrated that children’s home participation frequency and involvement did not change significantly over this time period [5]. One potential explanation for this finding is related to the impact the home environment may have on caregivers’ dissatisfaction with their child’s participation. Prior literature has consistently found that the home environment is associated with increased participation [5, 21], and our prior work with these data found that the majority (89%) of caregiver strategies focus on tailoring the child’s environment to promote their child’s participation at home following PICU discharge [19]. Therefore, it is possible that while the child’s participation may not change in the first 6 months post-PICU, caregivers may have adapted the home environment to make it more supportive for the child to resume participation in activities [29], thereby influencing their dissatisfaction with their child’s participation over time [24].

Another possible explanation for this finding of change in caregiver dissatisfaction relates to caregiver expectations for their child’s participation. Caregivers’ expectations for their child vary in response to multiple factors, including their child’s age and abilities [30, 31]. This is mirrored in the finding that the child’s increased functional performance was associated with higher levels of dissatisfaction, suggesting that the more independent the child is in performing discrete tasks, the more the caregiver may expect of their child’s participation in the home and thus the more they report being dissatisfied with their child’s home participation change.

The interaction of having strategies and having received PICU-based rehabilitation services was associated with less caregiver dissatisfaction with their child’s home participation, more so than any other variable in the model. Rehabilitation services often involve engaging caregivers in the defining service need, determining priority goals, and developing strategies for goal attainment. It is therefore possible that caregivers who received PICU rehabilitation services were better equipped to facilitate their transition home and thus were more satisfied with their child’s participation in the home. Prior analyses of data on this cohort has revealed a similar finding, in that the interaction of having participation-focused strategies and receiving PICU rehabilitation services was significantly associated with decreased caregiver stress [18], suggesting that this combination is associated with improved child and family outcomes.

Unfortunately, only 54% of our sample received any PICU-based rehabilitation services. This finding is consistent with prior literature suggesting that less than half of this population receive PICU rehabilitation services as a part of usual care [32–34]. Recent randomized control trials have found it feasible and safe to increase PICU-based rehabilitation services [35, 36], and acute rehabilitation is the only intervention that has been identified thus far for improving physical function in critically ill patients [37, 38]. Inclusion of rehabilitation services in the care of PICU patients allows for personalized strategic plans to be implemented for patients and families that can facilitate a smoother transition home and improve child and family outcomes.

Caregiver stress was associated with higher levels of dissatisfaction with their child’s home participation. This is consistent with prior literature that found caregiver stress is associated with other poor outcomes for children, such as hospital length of stay for the PICU population and for poor recovery in other populations [4, 22, 39]. It is also possible the stress itself makes it difficult for caregivers to be satisfied with their child’s current participation levels, or that their stress is a result of their child’s participation not meeting their expectations.

Additionally, caregiver dissatisfaction with their child’s participation prior to the PICU admission was significantly positively associated with their dissatisfaction post-PICU. This is important to note given the heterogeneity of the PICU population, with many children admitted having a pre-existing chronic condition and impaired functioning at baseline [1]. Considering the child and family’s pre-PICU status may help to direct PICU-based resources to improve functional outcomes for those who are most vulnerable.

The home environment is a complex and dynamic contextual factor that influences a child’s participation in daily life activities [14]. Relationships and attitudes of others, activity demands, and the physical layout and sensory qualities within the home were significantly associated with the caregivers’ dissatisfaction with participation, but access to resources for supporting their child’s home participation was not. This finding aligns with prior studies involving children with physical disabilities, in which limited resources (e.g., time, money), were not associated with children’s participation in leisure activities [40], and highlights the importance of assuring that caregivers are equipped with a broad range of participation-focused strategies for altering their home environment to support their child’s participation post-PICU [6, 13].

This study had several limits to generalizability. First, limited demographic data (e.g., socioeconomic status, race/ethnicity) resulted in leveraging data from the PEM environment section to estimate whether caregivers had adequate resources (time, money, information, and supplies) to support their child’s home participation. These PEM data on resources are reported when describing the study sample, and resources were significantly associated with caregiver dissatisfaction with participation. Second, as in most longitudinal studies, our sample had some missing data. We approached this issue with our statistical approach of generalized estimating equation modeling and by only including participants with data on the outcome variable at one or more of the three time points. However, post-hoc analyses found that those without data on caregiver dissatisfaction had significantly higher dissatisfaction at baseline. Thus, results should be interpreted with caution as it may be biased towards those with higher levels of functioning at baseline. Third, data on rehabilitation service use outside of the PICU were not collected but may impact caregivers’ dissatisfaction with participation at 3 and 6 months post-PICU, as well as important predictors (e.g., post-PICU rehabilitation may equip caregivers with participation-focused strategies and resources). Future studies examining pediatric critical survivorship would benefit from capturing more detailed information on rehabilitation services used during and post-PICU (e.g., intensity of services and strategies developed in sessions).

## Conclusion

More children are surviving critical illness with newly acquired impairments and there is need to improve their functional recovery, thereby improving the quality of survivorship for children and their families. Results highlight the importance of PICU-based rehabilitation services equipping families with participation-focused strategies to ease their transition home. When developing participation-focused strategies with caregivers, rehabilitation professionals should anticipate common areas of dissatisfaction in personal care management, getting clean, mealtime, household chores and homework. Rehabilitation professionals should focus on creating a supportive home environment for the child, by addressing the physical layout and sensory qualities of the home, demands of the activity, and the attitudes and relationships of family members and professionals who interact with their child in the home.

## Supplementary information

**Additional file 1.**

## Data Availability

The datasets generated and/or analyzed during the current study are not publicly available due to not having obtained informed consent for publication of the dataset containing clinical data at the point of recruitment. We thank Research Open Access Publishing (ROAAP) Fund of the University of Illinois at Chicago for financial support towards the open access publishing fee for this article.

## Abbreviations

PICU: Pediatric intensive care unit
PEM: Participation and Environment Measure
YC-PEM: Young Children’s Participation and Environment Measure
PEM-CY: Participation and Environment Measure for Children and Youth
IQR: Interquartile range
