# Supplementary material for: Caregiver dissatisfaction with their child’s participation in home activities after pediatric critical illness
Source: BMC Pediatr. 2020 Sep 2;20:415. doi: 10.1186/s12887-020-02306-3 (PMC7466418; doi:10.1186/s12887-020-02306-3)
Supplement: Supplementary file 1 — Additional file 1. [file 12887_2020_2306_MOESM1_ESM.docx]

Appendix.

**Table.**  Measurements for Child, caregiver, and home environmental factors post-PICU

|  | 3 months post-PICU | 6 months post-PICU |
| --- | --- | --- |
|  | N = 116 | N = 109 |
| Child Factors |  |  |
| Functional performance, median (IQR) | 46.8 (2.8-79.9) | 54.0 (2.8-84.2) |
| Home participation involvement, median (IQR) | 4.0 (3.3-4.4) | 3.9 (3.5-4.4) |
| Caregiver Factors |  |  |
| Caregiver stress difficulty, median (IQR) | 95.5 (67.3-126.8) | 90.5 (59.8-119.5) |
| Caregiver has participation-focused strategy, N (%) | 77 (66%) | 71 65% |
| Home Environmental Factors: |  |  |
| Attitudes and relationships | 100% (100-100%) | 100% (100-100%) |
| Activity demands | 100% (33-100%) | 100% (50-100%) |
| Resources | 100% (80-100%) | 100 (80-100%) |
| Physical layout and sensory qualities | 100% (50-100%) | 100% (100-100%) |

IQR = Interquartile range presented as Q1-Q3; Functional performance = Daily Activities domain of the Pediatric Evaluation of Disability Inventory – Computerized Adaptive Test; Caregiver stress difficulty = Pediatric Inventory for Parents; Participation involvement, participation-focused strategy, and home environmental factors (support) = Participation and Environment Measure; higher number indicates more involvement and environmental support
